# Supplementary material for: Are carbon emissions trading and green financial instruments synergistic? -Comprehensive quantitative research based on content analysis
Source: PLoS One. 2024 Mar 7;19(3):e0298601. doi: 10.1371/journal.pone.0298601 (PMC10919601; doi:10.1371/journal.pone.0298601)
Supplement: S1 Data — (ZIP) [file pone.0298601.s001.zip › Data/地级市Z_F工作报告.zip!/┴╔─■/┤≤┴1⁄4╩╨/└┤╫╘╣1⁄2╓┌║┼í╛┬φ┐╦╩2╛▌═°í┐_2011.docx]

各位代表：

　　现在，我代表市政府向大会报告工作，请予审议，并请市政协各位委员提出意见。

　　一、2010年和“十一五”时期政府工作回顾

　　刚刚过去的一年，是我市实施东北老工业基地振兴和辽宁沿海经济带开发开放战略、推进科学发展新跨越取得重大成就的一年。在省委、省政府和市委的正确领导下，市政府团结带领全市人民，抢抓机遇，奋力拼搏，圆满完成了十四届人大三次会议确定的各项任务。

　　预计全市实现地区生产总值5150亿元，增长15.2%；完成地方财政一般预算收入500.8亿元，增长25.1%；完成全社会固定资产投资5008亿元（常规口径），增长53%；实现社会消费品零售总额1630亿元，增长17.8%；实际使用外资100.3亿美元，增长66.7%；城镇居民人均可支配收入21300元、农民人均纯收入12120元，分别增长12%、13%；城镇登记失业率2.7%。我市主要指标增速位居全国副省级城市前列，年初确定的“三个五”目标胜利实现了！

　　（一）大力推进全域城市化，区域发展新格局加快形成

　　编制完成《大连市城市总体规划》、《大连建设区域核心城市规划》以及“三核两翼”港口布局规划，全域城市化战略实施规划和普湾新区、长山群岛等重点区域规划编制正在加快进行，“四大组团”空间布局、功能定位进一步明晰，港口、经济区和城市联动发展、互相促进的良好态势开始显现。制造业布局向北转移、基础设施向北延伸、生产要素向北集聚，城市空间沿黄渤两海、沈大和丹大两线加速拓展。

　　实施新市区管理体制改革，整合成立了金州新区、普湾新区，大幅拓展保税区发展空间，新市区“三足鼎立”框架基本形成。甘井子区综合配套改革不断深化。长兴岛临港工业区升级为国家级经济技术开发区和省综合改革试验区，庄河港正式对外开放，成立了长山群岛旅游避暑度假区。新增辽宁沿海经济带重点发展和重点支持区域8个，形成了多点支撑、全域开发开放的新局面。

　　区域开发建设全面提速，产业特色和集聚效应日益强化。主城区以服务业为主的经济结构基本形成，钻石海湾、旅顺南路软件产业带、大连生态科技创新城等重点区域建设全面展开，绿地中心等一批重点项目开工。金州新区“十大现代产业园区”、保税区汽车物流城、普湾新区起步区规划建设全面启动，大众变速箱二期、低碳与绿色建筑产业基地等项目相继落地，中船重工核电装备等项目陆续开工。花园口引进融德特种材料等国际领先企业，庄河国家生态工业示范园、长山群岛旅游避暑度假区综合体项目开工建设。长兴岛STX造船、恒力石化和瓦房店轴承城等项目建设加快。

　　（二）积极促进投资消费出口协调增长，经济运行质量明显提高

　　组织开展 “招商年”活动，加大境内外招商引资力度，实际使用外资增速位列全国副省级城市首位，浦项板材等一批重大外资项目落地进资；引进内资1020亿元，增长57.7%。实际使用外资和引进内资均实现了历史性突破。

　　狠抓重大项目建设，85个重点实施项目相继开工，哈大客运专线（大连段）、国际会议中心主体工程竣工，城市地铁、机场三期扩建、新机场物流园、新火车站、普湾大桥、长山大桥等项目开工建设。完成房地产投资768亿元，增长32.7%。投资对经济增长起到了重要拉动作用。

　　深入实施“促消费、保增长”10项措施，举办一系列大型主题促销活动。大连国际花卉等10大类商品市场和恒隆广场等高端商业项目开工建设，大商集团销售额突破800亿元，中山区入选“中国商业名区”。全市商品房销售额增长18.8%，汽车销售额增长35%。驼山汤海休闲度假区等重大旅游项目开工，国际服装节暨狂欢节、国际啤酒节等18项节庆活动拉动效应明显，实现旅游总收入558.4亿元，增长16.3%。消费需求进一步释放。

　　完善促进出口各项措施，积极开拓市场，引导企业扩大出口规模、提高出口产品质量。外贸出口260亿美元，增长25%。高新技术产品、机电产品出口分别增长53%和37%。境外投资增长97%。对外贸易促增长、调结构成效显著。

　　（三）加快推进产业升级，经济结构不断优化

　　优势产业加速升级，奇瑞汽车等重大项目建设进展顺利，国内首台、具有自主知识产权的一重（大连）百万千瓦级核反应堆压力容器发运、中远船务深水钻井船顺利下水、大橡塑20万吨大型挤压造粒机组试车成功。装备制造、石化、造船、电子信息等千亿级产业集群加快形成。完成规模以上工业增加值2300亿元，增长23.5%。

　　加快培育战略性新兴产业，英特尔芯片、皿能光电等项目投产，中科院大连科技创新园、大连检测科技园、海洋经济产业园等示范工程建设进展顺利，IBM中国区总部落户大连。完成新产品开发2650项，增长20%。重工•起重在国内率先研制成功5兆瓦海陆两用风电机组样机，“六轴7200千瓦大功率交流传动电力机车研发应用”获国家科技进步一等奖。规模以上工业高新技术产品增加值增长26.2%。我市跻身首批“国家高技术服务产业基地”，成为国家知识产权工作示范城市和 “三网融合”试点城市。

　　组建国有资源投资公司，重工•起重、瓦轴、金重股权多元化改革完成，染化集团改革和搬迁改造加快。完成海外并购项目18个。设立中小企业创业发展信贷风险补偿专项资金3亿元，成功承办第四届中国中小企业节。民营经济增加值增长18.5%。

　　大力发展设施、休闲等“八大农业”，创建都市型现代农业示范区16个。战胜低温、寡照、多雨等不利影响，粮食总产达到30亿斤，农林牧渔及服务业总产值增长7%左右。都市型现代农业呈蓬勃发展之势。

　　（四）完善提升城市功能，环境品质进一步改善

　　长兴岛30万吨原油码头、旅顺新港扩建等工程建设加快，港口货物吞吐量突破3亿吨、集装箱吞吐量526万标箱，大连机场旅客吞吐量超千万人次，保税港区辐射带动功能进一步增强。大窑湾铁路集装箱中心站、牡丹江穆陵内陆港竣工。东北亚煤炭交易中心正式运营。星海湾金融城一期投入使用，欧力士中国总部项目开工，渤海银行等19家金融机构落户大连，新建3家村镇银行和20家小额贷款公司。实现多渠道融资2200亿元，其中新增各项贷款余额1423亿元，增长26%。发起设立股权投资引导基金和辽宁沿海经济带发展基金，大连港集团等9家企业成功上市。

　　金州至普湾新区城际铁路、机场快速路、南部滨海大道开工建设，大连湾疏港高速公路、旅顺南路快轨、胜利路东段等工程有序推进，长兴岛疏港高速公路、疏港铁路通车。完成国省干道、县乡级公路大中修改造248公里，新建农村公路206公里。新开、延伸延时城市公交线路15条，更新公交车323辆。完成城建“五个一”和“三个一”工程112项。改造旧燃气管网158.2公里、城市供水旧管网185公里。大伙房水库输水入连南段工程完工。西部通道南段立交桥、东北路南段立交桥和联合路拓宽等一批道桥工程竣工。

　　严格控制新上高耗能项目，804台节能与新能源汽车示范运行，万元GDP综合能耗下降4.3%以上。加强大气环境综合整治，主要污染物减排3%以上，城市空气质量优的天数再创有监测历史以来最好水平。完成河道生态建设86条（段），治理河堤450公里。营城子、虎滩新区污水处理厂建成，城市污水集中处理率91.9%。实施造林绿化十大重点工程，植树1.04亿株，造林30万亩。城市绿化覆盖率45%，人均公共绿地13平方米，我市成为全国造林绿化模范市。

　　（五）着力保障和改善民生，社会建设全面推进

　　市政府为民办15件实事全面完成。实现城镇就业16.1万人。城市4.5万名无职业老年居民纳入社会保障范围，城镇居民基本医疗保险实行市级统筹。连续第6年提高企业退休人员养老金。临时救助制度实现城乡居民全员普惠。新建城乡社区养老服务中心80个。全市慈善基金总量3.4亿元。新增供热面积485万平方米，煤气配套历史遗留问题全部得到解决。采取货币补贴方式安排经济适用住房8000户，为市内四区4294户家庭发放廉租住房补贴，安排建设公共租赁房1500套，住房公积金支持11万户中低收入家庭改善住房条件。完成生态移民搬迁1025户，对31个低收入村实施扶贫开发。新建农村安全自来水工程101项。实施贫困残疾人家庭无障碍改造3100余户。沙河口区和中山区建成“国际安全社区”。法律援助覆盖面进一步扩大。

　　新建19所农村乡镇中心幼儿园，提高了城区公办幼儿园入园率。为219所农村中小学装备教育信息化“班班通”设备。实施农村困难家庭学生免费职业教育。国家基本药物制度全面实施，启动9类国家基本公共卫生和6项重大公共卫生服务项目，新型农村合作医疗参合率提高到99%。开工建设新儿童医院、妇产医院、新华医院。“健康家庭促进计划”服务12.9万户家庭，6项计生奖励扶助政策惠及36万人次。汉墓博物馆和宏济大舞台建成投入使用，农村公共文化服务工程全面完成。体育新城、金石国际运动中心建设加快，群众体育、竞技体育取得新成绩，大连籍运动员在广州亚运会、亚残运会上分别夺得12枚和4枚金牌。

　　进一步完善治安防控网络，大力整治突出治安问题，努力维护社会平安稳定。食品药品安全整治力度加大，“食品安全城市”建设深入推进。进一步加强对重大工程、交通运输和危险化学品生产经营的安全监管，切实做好应急管理。成功扑救“7•16”中石油原油储备库输油管线爆炸火灾，取得了海上和岸线清污的全面胜利，充分展现了全市人民众志成城、顽强拼搏、攻坚克难、勇于奉献的精神风貌。

　　加强重点领域专项审计和执法监督检查，切实规范行政执法行为。深化行政审批制度改革，市本级行政许可项目网上审批100%，行政效能进一步提高。深入推进政务公开，行政权力运行制度建设试点工作取得成效，政府公信力进一步增强。自觉接受人大及其常委会的法律监督、工作监督和政协民主监督，认真执行人大决议，坚持重大事项向人大常委会报告和向政协通报制度，积极吸纳各民主党派、工商联、无党派人士建言献策的意见和建议，自觉接受群众和舆论监督。办结人大代表建议926件、政协提案603件，满意率达95%以上。

　　民族、宗教、外事、侨务、对台、人防、气象、防震减灾、档案、双拥共建、民兵预备役等工作取得了新成绩。

　　各位代表，2010年是“十一五”规划的收官之年。五年来，我们经历了一系列大事、喜事和难事，有效应对国际金融危机巨大冲击，战胜“3•4”风暴潮等重大灾害，成功承办两届夏季达沃斯年会，获得“中国最佳旅游城市”等殊荣，率先全面振兴取得重大历史性成就。

　　——综合经济实力大幅跃升。五年间，地区生产总值年均增长16.3%，财政收入年均增长27%，分别比“十五”时期提高2.3和12.7个百分点。2010年人均GDP突破1.1万美元。全社会固定资产投资是“十五”时期的4.7倍。经济发展速度与质量效益全面提高，走在全国副省级城市前列。

　　——现代产业体系加快形成。五年间，传统产业改造升级取得突破性进展，“四大基地”集聚效应彰显，战略性新兴产业和现代服务业快速发展。万元GDP能耗下降20%以上，污染减排任务超额完成。大力实施创新驱动战略，科技进步贡献率提高15个百分点。大连成为承接国际国内产业转移的热点地区之一，国际竞争力大幅提升。

　　——城市综合功能日趋完善。五年间，完成城建总投资是“十五”时期的3倍。港口货物、集装箱和机场旅客吞吐量分别年均增长12.6%、14.4%、14.6%。连接海内外、沟通沿海地区、辐射东北腹地的物流集疏运体系加快形成。新设金融机构65家，新增上市公司24家，上市期货新品种4个，各项金融指标年均增长20%以上。我市的吸纳辐射能力显著增强，核心龙头地位不断强化。

　　——改革开放步伐不断加快。五年间，政府机构改革、行政管理体制改革、国企改革成效显著，社会主义市场经济体制不断完善。累计使用外资是“十五”时期的2.4倍，100家“世界500强”企业在连投资，年进出口总额突破500亿美元。累计引进内资2699亿元。我市保持了在区域竞争中的优势地位，发展动力进一步增强。

　　——县域经济发展势头强劲。五年间，“一岛十区”及产业项目在县域布局和开工建设，现代农业发展和新农村建设加快。7个涉农区（市）县GDP年均增长18.4%，高于全市2.1个百分点；财政收入年均增长31.7%，高于全市4.7个百分点。北三市在全国百强县的位次持续提升，瓦房店市进入前20强。县域经济成为新的发展引擎，支撑作用日益凸显。

　　——人民生活质量持续改善。五年间，市县两级财政直接用于民生的支出801.6亿元。全市新增就业80万人，比“十五”时期增长18.3%。城乡居民收入年均分别增长12.2%、15.5%。社会保障体系进一步完善，社会事业加速发展，生态环境不断优化。市民得到更多实惠，幸福指数逐步提高。

　　五年来，我们紧紧抓住东北老工业基地振兴和辽宁沿海经济带开发开放等重大历史机遇，始终把发展作为第一要务，努力争创新优势，保持了经济社会又好又快发展；始终把经济结构战略性调整作为转变发展方式的主攻方向，加快“三个中心、一个聚集区”建设，经济质量和效益不断提高；始终把改革开放创新作为发展的不竭动力，努力构建有利于科学发展的体制机制，不断激发城市的生机活力；始终把维护发展群众利益作为工作的出发点和落脚点，千方百计造福民生，切实让人民群众共享发展成果。所有这些，是过去五年我市取得突出成就的重要经验，也是未来大连实现跨越发展的宝贵财富。

　　各位代表，成绩来之不易！这一切得益于省委、省政府和市委的正确领导，源于多年工作的厚积薄发，倾注了广大干部群众的心血和汗水。在此，我代表市政府，向全市人民，向各级人大代表和政协委员，向各民主党派、工商联、人民团体和无党派人士，向离退休老同志，向中省直各部门和兄弟省市驻连机构，向驻连部队和武警官兵，向关心支持大连建设发展的海内外朋友们表示衷心的感谢！

　　在肯定成绩的同时，我们必须清醒地看到，我市的发展还面临不少困难和问题：外需、资源和环境对经济发展的制约加剧，经济结构调整和发展方式转变任务艰巨，城乡居民收入增长不快，住房、物价、交通和城市管理等事关民生的问题仍较突出，政府自身建设、经济发展软环境仍需进一步加强和优化。对此，我们要在今后工作中着力加以解决。

　　二、“十二五”时期发展思路、主要目标与2011年重点工作

　　各位代表，未来五年是大连加快经济发展方式转变的攻坚期，也是大连建设区域核心城市、向东北亚重要国际城市迈进的重要机遇期。“十二五”时期全市经济社会发展的指导思想是：高举中国特色社会主义伟大旗帜，以邓小平理论和“三个代表”重要思想为指导，深入贯彻落实科学发展观，以科学发展为主题，以加快转变经济发展方式为主线，以推进全域城市化为载体，以持续改善民生为出发点和落脚点，以深化改革开放为动力，优化空间布局，提升城市功能，调整经济结构，增强创新能力，保护生态环境，加快社会建设，为率先实现老工业基地全面振兴、建设东北亚重要国际城市而努力奋斗！

　　“十二五”时期的主要目标任务是：

　　综合实力显著增强。到“十二五”期末，地区生产总值、固定资产投资翻一番，分别达到1万亿元。实现地方财政一般预算收入1000亿元。科技、教育、文化、人才、创新优势彰显，城市核心地位和龙头作用更加突出。

　　民生质量显著改善。五年新增城镇就业100万人，城乡养老保障和基本医疗保险制度实现全覆盖，多层次的社会救助体系更加完善，实施保障性安居工程确保中低收入家庭住有所居，城乡居民收入与经济发展同步增长，人民群众生活水平和幸福指数明显提高。

　　社会建设显著加强。教育事业优先、均衡发展，公共卫生服务体系更加健全，文化事业和产业大发展大繁荣，“四馆四中心”建成投入使用，基本公共服务均等化加快实现。社会管理机制创新，民主法制更加完善，“平安大连”、“和谐大连”建设迈上新台阶。

　　经济结构显著优化。加快构建战略性新兴产业引领、先进装备制造业支撑、现代服务业发达的新型产业体系。装备制造、石化、造船千亿级产业集群和世界级产业基地基本建成，软件和服务外包争创“中国第一、世界第一”。县域经济和都市型现代农业加快发展。具有国际竞争力的现代产业聚集区加速形成。

　　全域城市化显著加快。进一步完善“四大组团”空间布局、城市功能、产业发展和生态建设规划，产业布局、基础设施、生产要素和重大项目加速向北转移和聚集，多中心、组团式、链条化城市新格局三年初具规模，五年基本形成，城市化率达到75%以上。

　　城市功能显著提升。实现港口货物吞吐量5亿吨、集装箱吞吐量1000万标箱，空港旅客吞吐量1600万人次以上；基本建成东北亚重要的货物中转、区域分拨配送基地和多功能综合服务中心；加快建设现代金融市场、金融服务体系和特色金融功能区；新机场、新老市区跨海通道等标志性工程项目主体工程完成。“三个中心”战略地位基本确立。

　　生态文明显著进步。坚持低碳、绿色发展，单位地区生产总值能耗、水耗和二氧化碳排放大幅下降，主要污染物排放总量明显减少；城市、中心城镇垃圾和生活污水处理实现全覆盖；建设国家森林城市，全市林木绿化率50%以上；大力发展循环经济，推广再生资源回收利用和清洁生产，努力把大连建设成为环境优美、低碳健康的生态宜居城市。

　　改革开放力度显著加大。创新和完善主城区管理体制，深化新市区体制改革，构建新型一体化管理模式；推进重点领域和关键环节改革，支持企业做强做大，进一步激发科学发展、创新发展、和谐发展的动力和活力。全面提高开放型经济水平和城市国际化程度。

　　各位代表，大连“十二五”规划蓝图已经绘就，我们迎来了一个激情绽放、蓬勃发展的崭新时代！我们坚信，经过五年的艰苦努力，大连“三个中心、一个聚集区”和生态宜居城市建设一定会取得决定性进展，城市综合实力和竞争力一定会大大加强，人民群众一定会生活得更富裕、更幸福！

　　2011年是“十二五”规划的开局之年，市政府工作的总体要求是：认真贯彻党的十七届五中全会、中央经济工作会议、省市委全会精神，深入推进全域城市化，加快“三个中心、一个聚集区”和生态宜居城市建设，实现“十二五”高质量开局、高水平起步。主要预期目标是：地区生产总值增长15%；万元GDP综合能耗降低3.5%，主要污染物减排2%；地方财政一般预算收入增长15%；全社会固定资产投资增长20%；社会消费品零售总额增长15%以上；实际使用外资增长10%；引进内资增长20%；外贸出口增长10%；城镇居民人均可支配收入和农民人均纯收入分别增长13%；城镇登记失业率控制在3%以内。

　　（一）深入实施全域城市化战略

　　以更加坚定的信心和有力的措施深入推进全域城市化，辽宁沿海经济带重点发展和支持区域开发建设年内初具形象，城乡经济社会发展全面提速。

　　优化功能区发展格局。加强城市组团和功能区产业布局、基础设施、生态环保、公共服务的统筹协调，推进城乡区域一体化发展。各区域都要确立更高的发展目标，打造若干支撑全域跨越发展的强力引擎。主城区要进一步向高端化、集约化方向发展，重点推进钻石海湾、星海湾金融商务区、新机场沿岸商务区、甘井子“两城四区”、高新区“十大软件产业园”、旅顺“五城一都”建设，打造服务经济发达的现代化城市中心。新市区要坚持产业化、城市化、国际化联动，重点推进金州新区“十大现代产业园区”、普湾新区起步区、保税区汽车物流城建设，加快形成新的城市功能拓展区和经济增长极。黄渤海区域要加快产业和要素集聚，重点推进长兴岛临港工业区、花园口新材料产业基地、瓦房店沿海经济区、庄河“三区十园”、长山群岛旅游避暑度假区建设，打造现代产业集群和新兴城市群。

　　加快“三个中心”建设。深化和完善“三核两翼”港口规划，加快大窑湾、太平湾、栗子房、长兴岛、双岛湾等重点港区开发建设，拓展集装箱海铁联运网络、内陆干港和中转业务，探索建立现代港航高端服务业孵化基地。开辟新的国际、国内空中航线，加快建设东北亚国际枢纽机场。规划建设综合物流园区，建立港航物流产业发展基金，培育和引进知名物流企业。加快金融功能区和金融体系建设，设立和引进新的金融机构，增加期货交易新品种，加大企业上市力度，推进跨境贸易人民币结算试点。

　　加大全域投资强度。以重点项目建设带动和促进社会投资，全年初步安排130个重点项目，总投资1.03万亿元。重点建设以普湾新区为核心，连接主城区、黄渤海区域的重大基础设施。创新投融资方式，积极引导金融机构加强信贷支持，多渠道筹措资金，优先保证重点在建、续建项目的资金需求，有序启动“十二五”规划重大项目建设。

　　（二）更加注重保障和改善民生

　　把保障和改善民生作为一切工作的出发点和落脚点，加大投入力度，办好15件实事，让人民群众共享发展成果。

　　切实增加城乡居民收入。深入实施富民工程，年内再次提高最低工资标准，及时发布工资指导信息，引导企业稳步提高职工工资水平。加大对低收入困难群体的补贴和救助力度，提高城乡低保标准，扩大覆盖范围。落实机关事业单位工资制度改革配套政策，平稳推行事业单位绩效工资制度。创造条件增加居民财产性收入和经营性收入。

　　千方百计稳定和扩大就业。实施更加积极的就业政策，全市实现城镇就业18万人。深化国家级创业型城市创建工作，充分发挥创业公共实训基地功能，以创业带动就业。继续实施高校毕业生储备和“三支一扶”计划，鼓励高校毕业生到城乡基层就业。加大就业困难群体就业援助力度，继续实施特别职业培训计划，提高劳动者就业能力。

　　不断完善社会保障体系。继续提高企业退休人员养老金标准。全面启动新型农村养老保障体系建设，将符合条件的农村居民纳入养老保障范围。加快推进城乡一体化医疗保障体系建设，实现城镇职工医疗保险市级统筹和医保卡全市通用，提高城镇居民医保待遇标准，新农合最低筹资标准提高到每人每年230元。积极探索建立政策相衔接、对象全覆盖、标准相协调的城乡社会救助体系。强化老龄工作，加快老年人社会服务体系建设。深化法律援助，做到应援尽援。

　　加快城乡保障性住房建设。采取政府引导、市场运作方式，加大公共租赁住房建设力度，努力解决中低收入群体住有所居问题。继续实施廉租补贴救助制度，对符合条件的申请家庭做到应保尽保。不断拓展住房公积金覆盖面，提高公积金使用效率。

　　努力解决群众切身利益问题。把稳定物价放在更加突出的位置，抓好新一轮“菜篮子”工程，确保粮、油、肉、蛋、菜等生活必需品市场平稳运行。继续推进城建“五个一”和“三个一”工程，维修主城区49条主干路、450条街巷路。下大气力整顿车辆乱停乱放现象，科学规划建设停车场，从根本上治理道路交通拥堵问题。改造旧燃气管网100公里、煤气进户支线2500户、旧住宅室内煤气管线3000户。改造室内供水旧管网5000户。新增供热面积240万平方米。切实做好弃管楼维修管理工作。完成生态移民搬迁1000户，对30个低收入村实施扶贫开发。新建农村安全自来水工程80项，解决10万人饮水安全问题。

　　（三）全面发展社会事业

　　坚持均衡与公益原则，加快建立完备的社会事业发展体系，强化公共服务能力，创新社会管理机制，促进社会和谐稳定。

　　繁荣发展文化事业和文化产业。加大文化事业投入，支持文艺院团建设，推进出版发行、文化创意、影视制作等文化主导产业发展。规划建设大型图书馆、博物馆、科技馆、城市规划馆、妇女儿童活动中心、青少年活动中心、职工活动中心、老年人活动中心等公共文化和服务设施。开展电影下乡、送戏下乡活动，繁荣文艺创作演出，丰富群众文化生活。加快体育新城等公共体育设施建设，市民健身中心投入使用。全面启动第十二届全运会比赛项目筹备工作，备战参赛第七届全国城市运动会，支持足球等优势项目创造更好成绩。

　　加强教育重点领域建设。实施加快教育改革和发展十大工程。新建12所城市公办幼儿园和28所乡镇中心幼儿园，扶持一批公益性、普惠性学前教育机构，切实解决幼儿入园难、入园贵问题。推进义务教育区域均衡发展，着力提升城乡教师的专业能力和师德水平。新建4所优质普通高中，全面完成中小学校舍安全工程，改善11所特教学校办学条件。整合优化资源，集中建设大连职业教育基地，扶持退役士兵培训基地建设。支持在连高校加快发展。

　　加快医疗卫生事业发展。推进医疗体制改革，深入落实国家基本药物制度，加强基层医疗卫生机构建设，努力解决群众看病难、看病贵问题。做好重点传染病防控、重大疾病监测报告，不断提高卫生应急处置能力。推进新卫生中心建设和老儿童医院改扩建。规划建设旅顺九龙湾生命健康城，推进大连国际医疗康复中心建设。深化“健康家庭促进计划”，促进人口长期均衡发展。

　　加强和创新社会管理。深入推进公安“三项建设”，加大对各类犯罪的打击力度，健全社会治安防控体系，进一步增强人民群众安全感。加强人民调解工作体系和机制建设，把矛盾纠纷化解在基层。充分发挥人民群众来访接待中心“一站式”调处化解功能，落实重大项目、重大决策信访稳定风险评估制度。切实加强安全生产，加快推进国家级危险化学品救援基地建设，坚决遏制重特大事故发生。开展打击侵犯知识产权和制售假冒伪劣商品专项行动，完善食品药品质量管理技术支撑体系。提升普法依法治理工作水平。巩固军政军民团结，再创国家和省双拥模范城。

　　继续做好民族、宗教、外事、侨务、对台、人防、气象、防震减灾、档案以及民兵预备役等工作。

　　（四）切实加快调结构转方式步伐

　　坚持把产业结构战略性调整作为经济转型的主攻方向，以增量调结构，以创新促升级，加快构建现代产业体系。

　　做大做强优势产业集群。全面实施工业“五项工程”，培育石化、电子信息、软件与服务外包等8个产值超千亿元的产业集群，轴承、精品钢材、生物医药等8个产值200—500亿元的产业集群。重点推进奇瑞汽车、一汽新能源客车、北车旅顺基地、恒力石化、中远造船、固特异轮胎、一重大连核电设备等在建项目，加快推动合资汽车整车、福佳MTO、延长集团长兴岛石化产业园等重大项目实施。

　　积极培育战略性新兴产业。在新能源、新材料、先进装备制造等10个领域，集中支持一批产业化项目和关键技术研发，推进航天软件研发基地等一批示范工程。加快“三网融合”试点工程建设，争取引进平板显示重大产业项目，抢占新兴产业发展的制高点。

　　优先发展现代服务业。推进网络动漫、科技研发、工业设计、创新服务等产业发展，不断壮大城区经济。启动“北方不夜港”工程，建设以旅顺、金石滩、长山群岛、金渤海岸等重点区域为依托的黄、渤海旅游经济带和北部温泉走廊，提升旅游产业的国际化水平。积极发展会展经济。推行信贷、网上消费等新兴消费方式，合理引导住房、汽车等消费。进一步完善城乡商品市场体系，深化“万村千乡”市场工程和农村中心镇现代商业建设，继续开展家电下乡和以旧换新，构建覆盖城乡的便民商业服务网络。

　　大力推进自主创新。以大连生态科技创新城、中科院大连科技创新园、大连检测科技园等重点园区为依托，加快建设综合科技服务基地，培育100家高成长性创新型企业。着力在先进装备制造、海洋与生物工程等领域开展关键技术研发和产业化应用。深入实施“区校一体化”，提升产学研合作水平。加强重点领域技术创新，培育一批产值10亿元以上高技术领军企业。办好“中国海外学子创业周”，大力引进、培养具有国际视野的创新型领军人才和中高级专业技术人才，加快建设人才强市。

　　（五）努力推动县域经济跨越发展

　　坚持多予、不取、放活，统筹城乡发展，深入推进农村工业化、城镇化和农业现代化，促进县域经济实力晋位争先。

　　加快发展都市型现代农业。做强做大优势农业、特色农业、精品农业，新发展设施农业10万亩、标准化设施小区1300个，新建都市型现代农业示范区20个。稳定粮食生产，进一步提高渔业、畜牧、水果、蔬菜、花卉等优势产业的规模和效益。积极推进农业产业化，促进农业龙头企业集聚发展。实施科教兴农，完善农业信息化和农产品标准化体系，夯实农业增效、农民增收的基础。

　　大力发展县域二、三产业。围绕县域重点工业园区建设，培育和做强做大一批核心竞争力强的产业集群。加快卫星城和重点城镇建设，积极发展县域现代服务业，做大住宅、旅游、商业和工业地产，促进市区、郊区、小城镇联动发展。

　　提高县域基础设施建设水平。统筹城乡基础设施建设，完善县域公共服务体系，通过科学规划实施村庄整体搬迁改造，推进乡镇“五通一净”、村屯“六化”工程，建设基础齐全、环境优美的新村庄、新农庄、新社区。加强农村道路建设，推进重点村屯通油路。完善水利基础设施，建设防汛应急工程35项，除险加固病险水库13座。

　　（六）不断深化改革扩大开放

　　深入实施区域管理体制、重点领域和关键环节改革，进一步拓展对外开放的广度和深度，营造助推科学发展新跨越的体制和环境。

　　加大改革攻坚力度。推进主城区、新市区管理体制创新，破除城乡二元结构，加快建立新型一体化管理模式。深化甘井子区综合配套改革，加快建立其他功能区与行政区融合发展新体制。制定政策措施，鼓励有稳定职业和收入的农民及其子女转为城镇户口，积极探索将进城务工人员纳入城镇住房保障体系。理顺市、县财政分配关系，增强市县财政保障能力。实施股权投资业发展计划，加快农村信用联社向农村商业银行转变的步伐。深入推进事业单位改革，全面实施岗位设置管理。加大企业兼并重组和海外并购力度，加快重工•起重、瓦轴、热电集团整体上市。健全公平公正的评价机制，完善社会诚信体系。

　　切实增强民营经济发展活力。加强引导、管理和服务，进一步支持民营企业扩大经营规模、提高技术和管理水平、转变发展方式，不断做强做大。进一步拓宽民营经济发展领域，清理整合涉及民间投资管理的行政审批事项，放宽企业出资限制要求，政府采购向民营企业全面开放。

　　提高对外对内开放水平。加大招商引资力度，坚持“引资”和“引智”相结合，提高利用内、外资的规模和质量。支持出口企业加强产品研发和自主创新，加快培育以技术、品牌、质量和服务为核心的国际竞争新优势。推动服务外包产业快速发展。深化国内区域经济技术合作。大力实施“走出去”战略，支持优势企业境外投资。办好第五届夏季达沃斯年会。推进长兴岛中日韩自由贸易试验区申报工作。

　　（七）大力提升基础设施和生态环境建设水平

　　围绕“两型社会”建设，进一步提高城乡规划、建设与管理水平，加快建设绿色、低碳、优美的生态宜居城市。

　　强化功能性基础设施建设。实施基础设施建设项目246项，总投资3200亿元。胜利路东段、旅顺南路快轨、振连路、大连湾疏港高速公路、皮口至炮台高速公路年内建成通车，机场三期扩建、新火车站、国际会议中心年内交付使用。加快推进城市地铁、金州至普湾新区城际铁路、长海机场扩建、红沿河核电二期、大连液化天然气接收站、大伙房水库输水入连等项目。启动新机场、丹大城际铁路、新老市区跨海通道、南部滨海大道西段连接线工程。加快构建国际化、现代化、一体化的城市基础设施体系。

　　加快“绿色大连”建设。全市投资100亿元，开展城乡大规模植树造林工程，加大山林植被保护力度，创建国家森林城市。办好第八届“中国城市森林论坛”。新改建公园34处、游园广场绿地32处。治理生态河道80条（段）。强化节能减排，加快淘汰落后产能，实施十大节能工程。以庄河国家生态工业示范园为载体，加快建设再生资源回收网络体系。　　做好环保模范城复查验收工作。加强海洋环境保护。

　　完善城市管理长效机制。启动数字化城管系统建设工程，强化城市管理执法和考核，实施环卫作业体制改革，下放城市街巷路管理权限。创新公共交通品牌服务。对主要路街和重点区域的市政设施和环境实施综合治理，改善老旧楼院环境，规范广告牌匾和建设工地秩序，营造整洁有序的市容市貌。

　　（八）进一步加强服务型政府建设

　　按照建设服务型政府的要求，深化管理创新，加强队伍建设，切实提高政府的执行力和公信力。

　　坚持依法行政。贯彻落实《国务院关于加强法治政府建设的意见》，切实提高政府依法行政水平。自觉接受人大法律监督、工作监督和政协民主监督，认真做好人大代表议案、建议和政协提案办理工作。推进行政决策科学化、民主化、法治化，全面加强行政执法规范化建设。

　　创新管理方式。完善社会管理职能，加强服务型政府和软环境建设，营造更加文明、热情、顺畅、高效的服务环境。改革投入机制，逐步增加财政资金投向公共服务领域的比重。加强应急管理与处置能力建设，加大应急物资、装备经费投入，最大限度地预防和减少突发事件造成的危害。全面整合行政服务资源，深入推进政务公开。提高电子政务应用水平，深入拓展电子监察功能。办好12345市民热线。

　　改进工作作风。始终保持奋发有为的精神状态，下大力气抓好工作落实。要深入基层和实际，直面困难，解决矛盾，善于把思路变成方案，把部署变成行动，把工作变成工程，出实招、干实活、求实效。凡事定了就干、干则必成。

　　加强廉政建设。全面开展加强权力运行制度建设工作，促进权力运行规范、透明、高效。深入抓好政风、行风建设，强化领导干部经济责任审计和重点建设项目资金跟踪审计，深化工程建设领域突出问题专项治理，严肃查处违法违纪案件，坚决纠治损害群众利益的不正之风。坚持勤俭办事、厉行节约，切实把有限的资金用到加快发展和改善民生上。

　　各位代表！新的蓝图引领新的征程，历史赋予的使命艰巨而光荣。让我们更加紧密地团结在以胡锦涛同志为总书记的党中央周围，在中共大连市委的领导下，凝聚全市人民的智慧和力量，振奋精神，开拓进取，扎实工作，在实现科学发展新跨越的伟大实践中再创辉煌！
